# Towards elimination of Chagas disease

**DOI:** 10.2471/BLT.25.294114

**Published:** 2026-05-11

**Authors:** Debbie Vermeij, Carlos M Morel, Claudia Chamas, Luiz V P Mendes, Andrea Silvestre-Sousa

**Affiliations:** a Oswaldo Cruz Foundation, Avenida Brasil, 4365 – Manguinhos, Rio de Janeiro, 21040-900, RJ, Brazil.

Chagas disease is a neglected tropical disease, caused by the protozoan parasite *Trypanosoma cruzi*. Six transmission routes for the disease exist: vectorial; through blood transfusion from an infected donor; through organ transplantation from an infected donor; oral; congenital; and accidental contamination.^1^ Chagas disease has two phases: an acute phase which is often mild or asymptomatic and generally lasts four to eight weeks, and a chronic phase. If untreated, infection is lifelong and an estimated 30–40% of people who are untreated will develop severe and sometimes life-threatening medical conditions, including cardiac, digestive and neurological or associated alterations, which might require specific treatment.^1^

Chagas disease is mainly found in the World Health Organization (WHO) Region of the Americas, where the large majority of the estimated 8 million people that are infected with the *T. cruzi* parasite reside.^2^ However, due to human mobility, Chagas is now present in nonendemic areas, both in and outside of the region. Each year, over 30 000 new cases and 12 000 Chagas disease-related deaths are reported. While considerable progress has been achieved in vector and other transmission control in the region, sustained efforts remain essential as vector-borne transmission persists in some endemic areas. Additionally, congenital (vertical) transmission has become increasingly relevant, with an estimated 9000 babies born with Chagas disease each year, affecting endemic as well as nonendemic areas due to migration, which has reshaped the epidemiological profile of the disease. However, due to the silent and neglected nature of the disease, these data are most likely underestimated.^1^^,^^3^

Symptomatic Chagas disease poses a substantial financial burden on health-care systems and societies, with an estimated 690 million United States dollars (US$) in health-care costs and US$ 8 billion in annual economic losses worldwide.^4^ However, despite the high morbidity and mortality of Chagas disease and the considerable associated economic burden, it has been estimated that less than 10% of people with the disease have been diagnosed and only about 1% receive etiological treatment.^5^

Over the past decades, the focus on Chagas disease has shifted from a vector-centred approach to a people-centred approach, with several international, regional and national initiatives to improve health service provision and access to diagnostics, treatment and comprehensive care, especially at the primary health-care level. In 2010, the International Federation of Associations of People Affected by Chagas disease, known as FINDECHAGAS, was founded to provide collective action and represent the voices of people affected by Chagas disease. Currently the federation includes more than 30 associations worldwide. In 2019, the seventy-second World Health Assembly established the World Chagas Disease Day to be observed on 14 April, to raise public awareness on the disease.^6^ In 2020, the first World Chagas Disease Day was observed, with the federation proposing the theme *Let’s make Chagas disease visible now*. The inclusion of Chagas disease in the annual World Neglected Tropical Disease Day and WHO’s *Ending the neglect to attain the sustainable development goals: a road map for neglected tropical diseases 2021–2030*^7^ complement these efforts.

Despite these efforts, important scientific and access gaps persist that, if addressed alongside social and environmental interventions, could accelerate and strengthen progress towards the elimination of Chagas disease as a public health problem.

First, improvements in the diagnosis of Chagas disease are needed. The diagnosis of chronic *T. cruzi* infection relies on serological tests. The Pan American Health Organization (PAHO) recommends using two serological assays that identify different antigens. If test results are inconclusive or conflicting, a third serological technique, with a different antigenic principle, should be employed. This diagnostic approach demands considerable time, specialized laboratory infrastructure and expertise, which are often unavailable in many remote and inaccessible regions, resulting in both delayed diagnosis and numerous undiagnosed cases. Over the past two decades, commercially available rapid diagnostic tests have been developed, which represent user-friendly diagnostic tools that typically yield results within 30 minutes. They do not need electrical equipment, are storable and remain functional at room temperature. Recent studies advocate for the possibility of implementing rapid diagnostic tests in health-care settings to offer a swift, dependable and conclusive individual diagnosis of chronic Chagas disease in remote regions.^8^

Diagnosis of acute Chagas disease is performed using either direct parasitological or, to a lesser extent, molecular methods. Real-time polymerase chain reaction has emerged as a promising technique for diagnosis, especially of congenital Chagas disease. Although molecular tests are more effective than the current diagnostic algorithm, their implementation in endemic countries is limited, primarily due to their complexity and high cost. New technologies are in development and could overcome some of these hurdles, particularly molecular point-of-care diagnostic tools like loop-mediated isothermal amplification; however, these technologies are not yet available for procurement.^9^ Enhancing access to diagnosis is imperative, particularly in Chagas disease-endemic areas where diagnostic facilities are scarce and/or inadequately equipped. Additionally, a pressing need exists to improve diagnostic algorithms that fit the needs of specific populations such as Indigenous or remote populations.

Second, ensuring access to effective, efficient and safe antiparasitic treatment for *T. cruzi* infection is critical for elimination. At present, only two pharmacological agents, nifurtimox and benznidazole, are efficacious in combating Chagas disease and have demonstrated efficacy in mitigating its duration and clinical severity.^10^^,^^11^ Parasite elimination is facilitated particularly when administered early in the natural progression of the disease (that is, during the acute phase, which includes congenital transmission, or in reactivation in immunosuppressed individuals) and in select chronic cases, excluding those with advanced Chagas cardiomyopathy or specific clinical contraindications such as liver failure and pregnancy. However, both drugs can cause adverse reactions such as dermatological, neurological, gastrointestinal and psychiatric reactions, leading to treatment discontinuation in up to almost one third of adults.^1^^,^^12^

Consistent availability of either drug, both in adult and paediatric formulations, is an additional continuous challenge. Benznidazole is currently produced by three different laboratories; however, only one of these has its product registered at the PAHO Strategic Fund, an initiative to pool procurement for essential diagnostics and treatment in the Americas. The low number of producers leads to a monopoly, which greatly affects availability and price. Nifurtimox is currently produced by only one laboratory, which donates it to the PAHO Strategic Fund. The dependency on donations negatively affects availability.

Third, the lack of sustainable funding for research and development impedes the development of improved diagnostic and therapeutic technologies for Chagas disease. The most pressing areas of research are biomarkers to assess therapeutic response and disease progression; effectiveness and safety of symptomatic treatment for Chagas heart disease; innovative diagnostic algorithms that can incorporate diagnostic platforms suitable at point of care; treatment options involving new chemical entities; improved dosage forms for use in children and newborns; and safer treatment options for pregnant women, among others.^13^

People affected by Chagas disease have a right to timely diagnosis and effective treatment, and this right must be guaranteed globally and collectively. A pressing need exists to reposition Chagas disease as one of the priorities on the global political agenda to ensure elimination of the disease as an international public health problem by 2030. However, doing so will only be possible through collaboration between all relevant stakeholders, seeking new sustainable governance and sufficient funding for technological, production and implementation challenges. To this end, we propose holding a high-level meeting on Chagas disease at the United Nations General Assembly in 2026. This meeting should aim to deliver concrete outputs, including a time-bound political declaration accompanied by a costed global action plan with measurable targets for diagnosis, treatment, comprehensive care, vector control and congenital transmission control. Results should also include sustained financing, pooled procurement commitments, a coordinated research and development and local production agenda to address market failures, and a clear monitoring and accountability framework aligned with the WHO roadmap for neglected tropical diseases.

Lessons from other neglected tropical disease elimination efforts demonstrate that eradication is achievable with sustained political commitment, simplified tools and equitable access to treatment. The elimination of human African trypanosomiasis as a public health problem and progress towards eliminating lymphatic filariasis and onchocerciasis illustrate the effectiveness of coordinated regional strategies, integration into primary care, community engagement and sustained international financing.^14^ These experiences provide valuable lessons for accelerating progress towards Chagas disease elimination.^7^

Addressing structural determinants of transmission is equally important. The *WHO housing and health guidelines*^15^ emphasize that improving housing quality is a key intervention for preventing vector-borne diseases. Substandard housing remains a major risk factor for Chagas disease transmission in endemic regions, highlighting the need for intersectoral approaches that integrate health, housing and development policies.

Without rapid and coordinated action, the goal of eliminating Chagas disease as a public health problem by 2030 is unlikely to be achieved. Elevating Chagas disease on the global political agenda, strengthening regional leadership in Latin America and ensuring equitable access to essential diagnostics, treatment and care tools are critical to achieving elimination targets. The experiences in addressing other neglected tropical diseases demonstrate that elimination is possible; therefore, the time to act on Chagas disease is now.

## References

[1] de Sousa AS, Vermeij D, Ramos AN Jr, Luquetti AO. Chagas disease. Lancet. 2024 Jan 13;403(10422):203–18. 10.1016/S0140-6736(23)01787-7 38071985

[2] Chagas disease (also known as American trypanosomiasis). Geneva: World Health Organization; 2025. Available from: https://www.who.int/news-room/fact-sheets/detail/chagas-disease-(american-trypanosomiasis) [cited 2026 Mar 16].

[3] Updated estimate of Chagas disease in the endemic countries of the Americas 2018. Washington, DC: Pan American Health Organization; 2025. Available from: https://iris.paho.org/handle/10665.2/68811 [cited 2026 Mar 06].

[4] Lee BY, Bacon KM, Bottazzi ME, Hotez PJ. Global economic burden of Chagas disease: a computational simulation model. Lancet Infect Dis. 2013 Apr;13(4):342–8. 10.1016/S1473-3099(13)70002-1 23395248 PMC3763184

[5] Chaves GC, Abi-Saab Arrieche M, Rode J, Mechali D, Reis PO, Alves RV, et al. [Estimating demand for anti-Chagas drugs: a contribution for access in Latin America]. Rev Panam Salud Publica. 2017 Jun 8;41:e45. Spanish. 28614468 10.26633/RPSP.2017.45 PMC6612731

[6] World Chagas Disease Day. 14 April 2024. Geneva: World Health Organization; 2026. Available from: https://www.who.int/campaigns/world-chagas-disease-day/2024 [cited 2026 Mar 16].

[7] Ending the neglect to attain the sustainable development goals: a roadmap for neglected tropical diseases 2021–2030. Geneva: World Health Organization; 2020. Available from: https://www.who.int/publications/i/item/9789240010352 [cited 2026 Mar 16].

[8] Perez F, Vermeij D, Salvatella R, Castellanos LG, de Sousa AS. The use of rapid diagnostic tests for chronic Chagas disease: An expert meeting report. PLoS Negl Trop Dis. 2024 Aug 8;18(8):e0012340. 10.1371/journal.pntd.0012340 39116064 PMC11309409

[9] Schijman AG, Alonso-Padilla J, Britto C, Herrera Bernal CP. Retrospect, advances and challenges in Chagas disease diagnosis: a comprehensive review. Lancet Reg Health Am. 2024 Jun 20;36:100821. 10.1016/j.lana.2024.100821 39006126 PMC11246061

[10] Hasslocher-Moreno AM, Saraiva RM, Sangenis LHC, Xavier SS, de Sousa AS, Costa AR, et al. Benznidazole decreases the risk of chronic Chagas disease progression and cardiovascular events: a long-term follow up study. EClinicalMedicine. 2020 Dec 23;31:100694. 10.1016/j.eclinm.2020.100694 33554085 PMC7846661

[11] Rassi A Jr, Grimshaw A, Sarwal A, Sah R, Shah S, Agudelo Higuita NI, et al. Impact of antiparasitic therapy on cardiovascular outcomes in chronic Chagas disease. A systematic review and meta-analysis. EClinicalMedicine. 2024 Dec 24;79:102972. 10.1016/j.eclinm.2024.102972 39810938 PMC11732499

[12] Mendes FSNS, Perez-Molina JA, Angheben A, Meymandi SK, Sosa-Estani S, Molina I. Critical analysis of Chagas disease treatment in different countries. Mem Inst Oswaldo Cruz. 2022 Jul 8;117:e210034. 10.1590/0074-02760210034 35830002 PMC9273179

[13] Schijman AG, Alonso-Padilla J, Britto C, Herrera Bernal CP. Retrospect, advances and challenges in Chagas disease diagnosis: a comprehensive review. Lancet Reg Health Am. 2024 Jun 20;36:100821. 10.1016/j.lana.2024.100821 39006126 PMC11246061

[14] Zhao H, Xu T, Shen H. Global, regional, and national disability-adjusted life years and prevalence of lymphatic filariasis from 1990 to 2021: a trend and health inequality analysis based on the global burden of disease study 2021. PLoS Negl Trop Dis. 2025 Apr 29;19(4):e0013017. 10.1371/journal.pntd.0013017 40300002 PMC12040265

[15] WHO housing and health guidelines. Geneva: World Health Organization; 2026. Available from: https://www.who.int/publications/i/item/9789241550376 [cited 2026 Mar 16].

